# Developmental landscape and asymmetric gene expression in the leaf vasculature of *Brassica rapa* revealed by single-cell transcriptome

**DOI:** 10.1093/hr/uhaf060

**Published:** 2025-02-26

**Authors:** Xinlei Guo, Jingping Yuan, Yuanyuan Zhang, Jian Wu, Xiaowu Wang

**Affiliations:** Henan Engineering Research Center of the Development and Utilization of Characteristic Horticultural Plants, School of Horticulture and Landscape Architecture, Henan Institute of Science and Technology, Xinxiang 453003, China; Henan Engineering Research Center of the Development and Utilization of Characteristic Horticultural Plants, School of Horticulture and Landscape Architecture, Henan Institute of Science and Technology, Xinxiang 453003, China; Henan Engineering Research Center of the Development and Utilization of Characteristic Horticultural Plants, School of Horticulture and Landscape Architecture, Henan Institute of Science and Technology, Xinxiang 453003, China; State Key Laboratory of Vegetable Biobreeding, Institute of Vegetables and Flowers, Chinese Academy of Agricultural Sciences, Beijing 100081, China; State Key Laboratory of Vegetable Biobreeding, Institute of Vegetables and Flowers, Chinese Academy of Agricultural Sciences, Beijing 100081, China

## Abstract

Leaf vasculature not only acts as a channel for nutrients and signaling information but also influences leaf morphology. It consists of several distinct cell types with specialized functions. Cell type-specific characterizations based on single-cell RNA sequencing technology could aid in understanding the identities of vascular tissues and their roles in leaf morphogenesis in *Brassica rapa*. Here, we generated a single-cell transcriptome landscape of the Chinese cabbage leaf vasculature. A total of 12 cell clusters covering seven known cell types were identified. Different vascular cell types were characterized by distinct identities. The xylem parenchyma and companion cells exhibited an active expression pattern of amino acid metabolism genes. Tracheary elements and sieve elements were enriched in many genes related to cell wall biosynthesis, and the phloem parenchyma was enriched in many sugar transporter-encoding genes. Pseudo-time analyses revealed the developmental trajectories of the xylem and phloem and the potential roles of auxin and ethylene in xylem development. Furthermore, we identified key candidate regulators along the differentiation trajectory of the sieve elements and companion cells. Most of the homoeologous genes in the syntenic triads from the three subgenomes showed asymmetric gene expression patterns in different vascular cell types. Collectively, our study revealed that Chinese cabbage leaf vasculature cells had highly heterogeneous transcriptomes, providing new insights into the complex processes of leaf vasculature development in *B. rapa* leafy vegetables and other *Brassica* crops.

## Introduction

During the evolutionary process from aquatic to terrestrial environments, plants produced an important transport system, vasculature, which enabled them to adapt to terrestrial environments through nutrient exchange between organs and the support of mechanical strength [1–3]. Leaf vasculature, the vascular system of the leaves, comprises two functionally distinct conductive tissues, namely the xylem, which is responsible for transporting water and mineral elements, and the phloem, which is responsible for transporting photoassimilates. The xylem, located on the adaxial side of the leaf, includes tracheary element (TE) and xylem parenchyma (XP), while the abaxial phloem can be divided into sieve element (SE), companion cell (CC), and phloem parenchyma (PP), which have been well characterized using microscopy [4]. The leaf vasculature not only acts as a channel for nutrients and signaling information but also influences leaf morphology when organized into different architectural networks [5]. The development of vascular tissue and the morphogenesis of leaf shape are influenced by adaxial–abaxial polarity genes. Mutations in these genes result in the loss of lamina flattening and altered leaf phenotypes, such as curly, crinkly, twisted, rolled, or shrunken leaves [6].

*Brassica rapa* is an important vegetable crop that contains different subspecies that have been domesticated with diverse leaf morphotypes, e.g. the leafy head of Chinese cabbage, flat leaves of pak choi, and downward-curling leaves of Wu tacai [7]. Compared to *Arabidopsis* and pak choi, the proportion of vasculature in the entire Chinese cabbage leaf is remarkably higher, and the proportion increases rapidly during the folding and heading stages [8]. During leafy head formation, the veins begin to curve and form the leaf midrib. Our previous domestication selection analysis found that two *BREVIS RADIX* (*BRX*) genes were under strong selection in leaf-heading *B. rapa* [7], indicating their role in leafy head formation. In *Arabidopsis*, *BRX* is preferentially expressed in the leaf phloem, and its mutation results in a curled leaf phenotype [3,9]. *OCTOPUS* (*OPS*), which encodes a polarly localized membrane-associated protein, is initially expressed in provascular cells, and its expression is restricted to the phloem cell lineage, regulating phloem differentiation and entry [10]. Zhang et al. found that *BrOPS* was localized in the leaf vasculature and regulated the expression of leaf polarity gene *ASYMMETRIC LEAVES 1* (*AS1*), thereby influencing the leaf curvature and heading shape of Chinese cabbage [11]. Therefore, the construction and comparison of gene expression profiles between different leaf vascular cells are crucial for understanding the regulatory mechanisms that control *B. rapa* leaf development and morphogenesis.

However, it is almost impossible to isolate different vascular cell types from thin leaves containing only a few layers of cells. To date, various techniques, including laser capture microdissection, fluorescence-activated cell sorting, and translating ribosome affinity purification followed by sequencing (TRAP-seq) have been developed to dissect and characterize individual cells or specific cell types from the roots, shoot apical meristems, or leaves of several plant species, such as *Brassica napus*, maize, and *Arabidopsis* [12–15]. However, these methods have many limitations, such as low throughput and technical challenges, biased sampling of different cell types, and dependence on stable genetic transformation system, all of which limits their application in non-model plants [16].

Recently, advances in single-cell RNA sequencing (scRNA-seq) technology have allowed high-resolution characterization of gene expression at the single-cell level. This technology has been applied to a wide range of tissues in plants, such as the roots of rice, the shoot tips of *Arabidopsis* and pea, the ears and anthers of maize, and the leaves of strawberry [17–22]. In addition, this technology can be used to explore developmental trajectories. For example, Zhang et al. constructed a differentiation trajectory for leaf vein tissue using scRNA-seq in *Arabidopsis* leaves and shoot apex [21]. In addition, Kim et al. identified seven vascular cell types in *Arabidopsis* leaves and found distinct developmental and metabolic features between PP and CC [3], providing marker genes and a reference for studying the vasculature of other cruciferous species. Three studies have revealed the single-cell transcriptome landscapes of poplar stems and reconstructed differentiation trajectories of the phloem or xylem [23–25]. Conde et al. revealed conserved and differentiated trajectories of vascular cell formation between *Populus* and *Arabidopsis* through single-nuclei transcriptome analysis of the shoot apex [26]. Using scRNA-seq, Sun et al. characterized the subgenome dominance of seven cell types in Chinese cabbage leaves and the transcriptional changes under high-temperature stress [27]. Despite the considerable progress of the vascular system in *Arabidopsis* and *Populus*, studies of leaf veins in *B. rapa*, especially at single-cell resolution, are still lacking. *Brassica rapa*, which belongs to the *Brassicaceae* family, differs from perennial woody poplars. Additionally, *B. rapa* underwent an extra whole-genome triplication after divergence from *Arabidopsis*, and as a result, many genes have multiple homologs and may be functionally divergent [28]. Although a single-cell transcriptome map of Chinese cabbage leaves has been constructed, leaf veins in previous studies have only been briefly described as a whole and not analyzed in detail. Therefore, it is essential to focus on the leaf vascular cells of Chinese cabbage, which would contribute to the study of the vascular tissues of other *Brassica* crops.

Here, we identified XP, TE, PP, CC, SE, and PC (procambium cell) cell populations from vascular cell populations of Chinese cabbage leaves by analyzing single-cell data. We also identified cluster-enriched genes and found distinct identities across vascular cell types. We reconstructed the developmental trajectories of the xylem and phloem and identified genes that changed significantly with the differentiation trajectory. The results not only provide the transcriptome landscape of leaf vascular cells at single-cell resolution in *B. rapa*, but also provide a framework to compare the transcriptomics of specific cell types in plant leaves between different developmental stages and species, facilitating an understanding of the complex processes of leaf development and morphogenesis in *B. rapa* and other *Brassica* crops.

## Results

### Seven vascular cell types were identified in *B. rapa* leaves

To enable scRNA-seq of Chinese cabbage leaves, young leaves were harvested at the rosette stage. Our samples had two batches, among which RL1 (Repeat leaf 1) belonged to batch 1 and two samples (RL2 (Repeat leaf 2) and RL3 (Repeat leaf 3)) belonged to batch 2. After being cut into strips, the leaf tissues were digested with an enzymatic solution to release the protoplasts. The collected protoplasts were subjected to the 10x Genomics platform for cDNA library preparation and scRNA-seq. As RL1 was the first sample, we aimed to detect as many genes as possible by increasing the sequencing depth. Although RL1 contained more sequencing data, the gene number detected was very similar among the three samples (Table S1).

After removing doublets, low-quality cells, and genes, 16055 high-quality cells (4317, 6090, and 5648 cells for RL1, RL2, and RL3, respectively) and 30214 genes were obtained. Using principal component analysis and unsupervised dimensionality reduction cluster analysis and visualization with the uniform manifold approximation and projection algorithm (UMAP), an obvious batch effect was observed between RL1, RL2, and RL3 (Figure S1A). Batch effects were removed using the R package ‘Harmony’, and analysis of the three samples revealed a reproducible data quality (Figure S1B).

**Figure 1:**
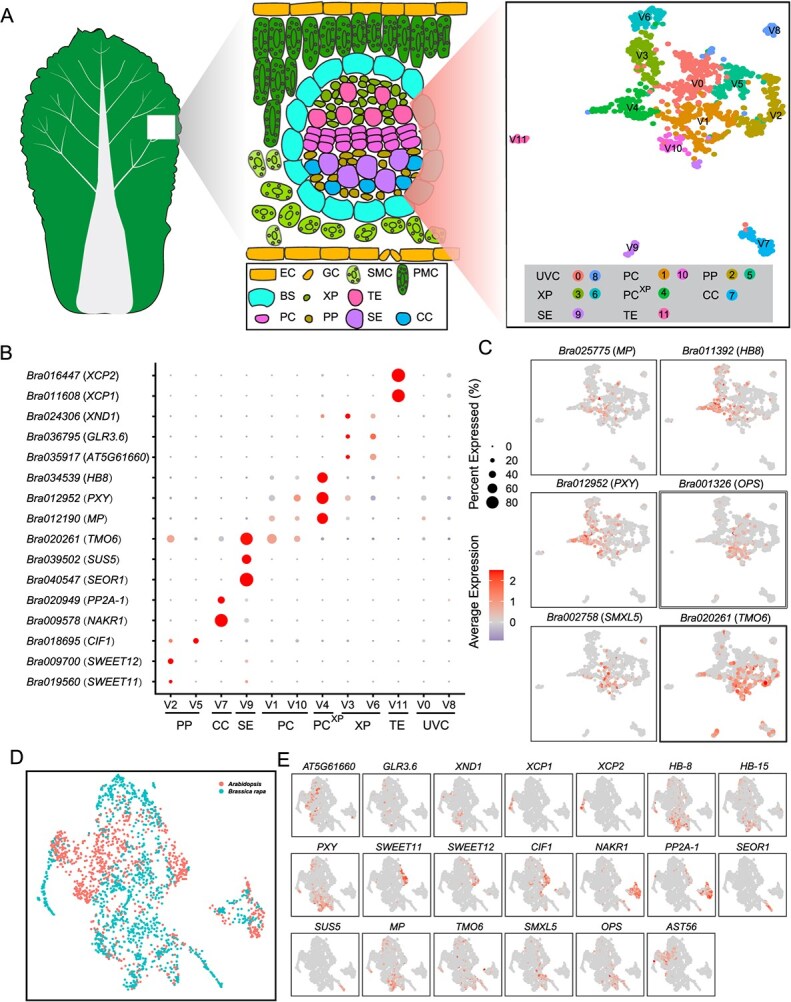
Cell type identification in the vascular tissue of Chinese cabbage leaves. **(A)** Visualization of 12 vascular cell clusters using UMAP. EC, epidermis; GC, guard cell; PMC, palisade mesophyll cell; SMC, spongy mesophyll cell; BS, bundle sheath; XP, xylem parenchyma; PC^XP^, procambium cells with features relating to xylem differentiation; TE, tracheary elements; PP, phloem parenchyma; CC, companion cells; SE, sieve elements; PC, procambium cell; UVC, unknown vascular cell. **(B)** Expression pattern of Chinese cabbage orthologs of the reported marker genes in *Arabidopsis*. **(C)** Expression characterization of key genes involved in the differentiation from cambium to xylem or phloem. **(D)** Integrated visualization of *B. rapa* and *Arabidopsis* cell clusters. **(E)** Expression characterization of marker genes in integrated data of *B. rapa* and *Arabidopsis*.

To comprehensively investigate the transcriptome of *B. rapa* leaf vascular tissue, we profiled the gene expression pattern of vascular tissue at single-cell resolution. Our previous scRNA-seq data from Chinese cabbage rosette leaves provided the data basis for this study [29] (Figure S2). A total of 1068 vascular cells identified in our previous study were selected and re-clustered, resulting in 12 clusters (V0–V11) (Figure S2 and Figure 1A). Due to the limited availability of vascular tissue marker genes for the non-model plant *B. rapa*, the orthologs of *Arabidopsis* marker genes were used to identify these clusters (Table S2). Using these marker genes, we identified seven cell types, namely XP, PC^XP^ (procambium cells with features relating to xylem differentiation), TE, PP, CC, SE, and PC (Figure 1B).

The xylem population included clusters V3, V6, and V11. In *Arabidopsis*, *AT5G61660*, *GLUTAMATE RECEPTOR 3.6* (*GLR3.6*), and *XYLEM NAC DOMAIN 1* (*XND1*) were highly expressed in the protoxylem and xylem [21,30,31]. Their orthologs *Bra035917*, *Bra017529*, and *Bra024306* were specifically highly expressed in clusters V3 and V6 (Figure 1B); thus, clusters V3 and V6 were assigned as XP. Cluster V11 was defined as TE due to the specific expression of two TE marker genes (*XYLEM CYSTEINE PEPTIDASE 1* (*XCP1*) and *XYLEM CYSTEINE PEPTIDASE 2* (*XCP2*)) [32,33] (Figure 1B). The phloem cell population contained clusters V2, V5, V7, and V9. Cluster V2 was defined as PP because of the specific expression of two genes encoding sucrose efflux transporters (SWEET11 and SWEET12) [34] (Figure 1B). In *Arabidopsis*, *CELL WALL / VACUOLAR INHIBITOR OF FRUCTOSIDASE 1* (*CIF1*) was specifically expressed in PP and involved in carbohydrate metabolism and sugar signaling [3]. Cluster V5, which was adjacent to V2, showed a high expression level of *CIF1*; thus, V5 was also assigned as PP (Figure 1B). In addition, *NAKR1* and *PROTEIN PHOSPHATASE 2A-1* (*PP2A-1*) were mainly expressed in CCs [35,36], and their orthologs were mainly expressed in cluster V7, which was defined as CC (Figure 1B). SE-specific marker SIEVE-ELEMENT-OCCLUSION-RELATED 1 (*SEOR1*) and *SUCROSE SYNTHASE 5* (*SUS5*) [37–39] were specifically expressed in cluster V9 and were thus identified as SE (Figure 1B).

*MONOPTEROS* (*MP*) encodes a transcription factor (TF) that mediates vascular development and acts as a marker gene for vascular tissue formation, and it was mainly expressed in the cells adjacent to clusters V1 and V4 (Figure 1C). Moreover, clusters V4 and V10 were enriched with several key genes involved in cambium to xylem differentiation, including *PHLOEM INTERCALATED WITH XYLEM* (*PXY*) and *HOMEOBOX GENE 8* (*HB8*) (Figure 1C) [40,41]. Therefore, V4 was assigned to PC^XP^. In *Arabidopsis*, *SMAX1-LIKE 5* (*SMXL5*) was detected in the procambium and part of the phloem, where it acts as a cell-autonomous key regulator of phloem formation [42]. *TARGET OF MONOPTEROS 6* (*TMO6*) was highly expressed in the phloem-procambium boundary based on RNA *in situ* hybridization [43]. Their orthologs were preferentially expressed in cluster V1 (Figure 1C). *BrOPS* was preferentially expressed in the meristem between the xylem and phloem in *B. rapa* [11] (Figure 1C). Thus, V1 and V10 were assigned as PC. The cell types of the remaining two clusters, namely clusters V0 and V8, were unable to be identified due to the absence of known marker genes.

To verify the cell cluster annotation, a published scRNA-seq dataset from *Arabidopsis* leaf vasculature [3] was used for comparison with our results. We downloaded this scRNA-seq dataset, reconducted clustering and cell type assignment, and obtained 15 cell clusters, including guard cells, epidermis, mesophyll cells, bundle sheath, CC, PP, and xylem (Figure S3A and B). Three cell clusters (clusters 4, 8, and 12) (Figure S3A), which contained all vascular cells and bundle sheath, were extracted and reclustered, yielding six cell clusters (Figure S3C), and the major vascular cell types were detected except for TEs (Figure S3D). To confirm the cell type annotation, a set of one-to-one orthologs between *Arabidopsis* and Chinese cabbage were identified (Table S3). The integration of single-cell data showed that these vascular cells from both species clustered well together (Figure 1D). In addition, compared with *Arabidopsis*, our data also included TE cells from Chinese cabbage (Figure 1E). These results demonstrate the reliability of the cell type annotation in this study, providing a basis for a comprehensive study of the transcriptome characteristics and developmental processes of the leaf vasculature.

### Distinct transcriptome characteristics of XP and TE

**Figure 2:**
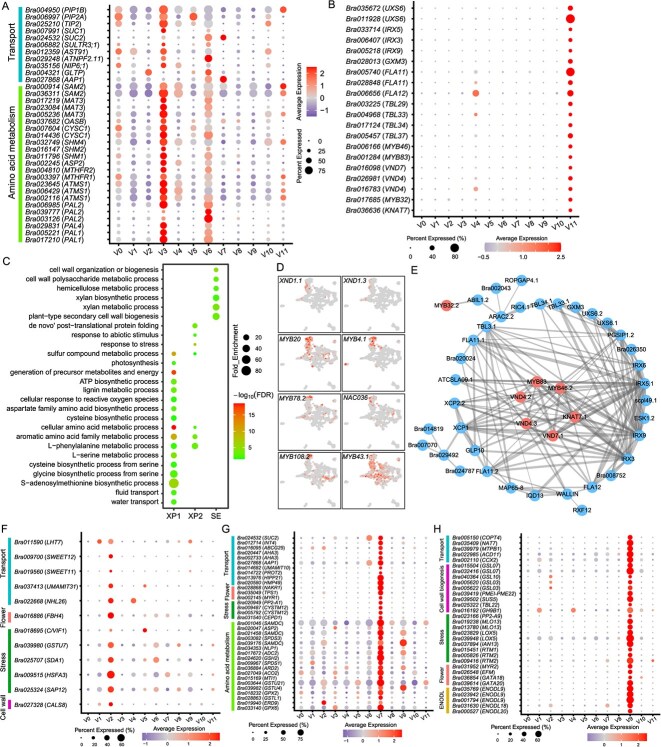
Heterogeneity of the xylem and phloem cell populations. **(A)** Expression characterization of genes related to transport and amino acid metabolism. **(B)** Expression characterization of genes related to xylan synthesis and secondary cell wall biosynthesis. **(C)** GO enrichment analysis of cluster-enriched genes in XP and TE. **(D)** Expression characterization of genes encoding TFs highly expressed in XP. **(E)** Putative protein–protein interaction network of these cluster-enriched genes in TE using STRING. The red dots represent the genes encoding TFs. **(F)** Expression characterization of genes highly expressed in PP. **(G)** Expression characterization of genes highly expressed in CC. **(H)** Expression characterization of genes highly expressed in SE.

Xylem parenchyma cells were distributed between TEs and were involved in water and minerals transport. XP cell clusters V3 (XP1) and V6 (XP2) were enriched with many transport protein-encoding genes, including aquaporins *PLASMA MEMBRANE INTRINSIC PROTEIN 1B* (*PIP1B*), *PLASMA MEMBRANE INTRINSIC PROTEIN 2A* (*PIP2A*), and *TONOPLAST INTRINSIC PROTEIN 2* (*TIP2*), sucrose transporter *SUCROSE-PROTON SYMPORTER 1* (*SUC1*) [44], sulfate transmembrane transporters *SULFATE TRANSPORTER 3;1* (*SULTR3;1*) and *SULFATE TRANSPORTER 91* (*AST91*) [45], glucosinolate importer *NRT1/ PTR FAMILY 2.11* (*ATNPF2.11*) [46], boron transporter *NOD26-LIKE INTRINSIC PROTEIN 6;1* (*NIP6;1*) [47], glycolipid transporter *PHOSPHOLIPASE-LIKE PROTEIN* (*GLTP*), and amino acid transmembrane *AMINO ACID PERMEASE 1* (*AAP1*) [48] (Figure 2A and Table S4). Additionally, gene ontology (GO) enrichment analysis showed that XP1 and XP2 were significantly enriched in genes involved in the cellular amino acid metabolic process (Figure 2A and Table S5). For example, XP1 was significantly enriched in the S-adenosylmethionine biosynthetic process, L-serine metabolic process, glycine biosynthetic process from serine, L-phenylalanine metabolic process, and cysteine biosynthetic process from serine (Figure 2A, C, and Table S5), which was supported by Kyoto Encyclopedia of Genes and Genomes (KEGG) enrichment analysis (Table S6). These findings imply that XPs are an important site of amino acid metabolism in *B. rapa*.

Unlike XP cells, cluster V11 (TE) enriched genes involved in xylan synthesis and secondary cell wall biosynthesis, such as UDP-glucuronic acid decarboxylase *UDP-XYL SYNTHASE 6* (*UXS6*) [49], cellulose synthase *IRREGULAR XYLEM 5* (*IRX5*) and *IRX3* [50,51], glycosyl transferase *IRX9* [52], methyltransferase *GLUCURONOXYLAN METHYLTRANSFERASE3* (*GXM3*), fasciclin-like arabinogalactan proteins *FLA11* and *FLA12*, and TRICHOME BIREFRINGENCE-LIKE (TBL) family genes (*TBL29*, *TBL33*, *TBL34* and *TBL37*) (Figure 2B). Additionally, GO enrichment analysis showed that cluster V11 (TE) was significantly enriched in cell wall organization or biogenesis, xylan metabolic process, and hemicellulose metabolic process (Figure 2C and Table S5). These results are consistent with the fact that TEs usually have an obviously thickened secondary cell wall.

TFs play critical roles in plant growth and development by promoting or repressing gene expression and protein synthesis. We found that several TFs, including *NAC* TFs *BrXND1.1*, *BrXND1.3*, and *BrNAC036*, and *MYB* TFs *BrMYB20*, *BrMYB4.1*, *BrMYB78.2*, *BrMYB108.2*, and *BrMYB43.1*, were preferentially expressed in XP (Figure 2D). In addition, we identified seven genes encoding TFs specifically expressed in TE, including *MYB* TFs *BrMYB46.2*, *BrMYB83*, *BrMYB32.2*, NAC-domain TFs *BrVND7.1*, *BrVND4.2*, *BrVND4.3*, and *KNOTTED-LIKE HOMEOBOX OF ARABIDOPSIS THALIANA 7* (*BrKNAT7.1*) (Figure 2B and Table S4). To explore the regulatory network during TE development, these TE-enriched genes were evaluated using STRING database to understand their interactions. Of these genes, 43 formed an interaction network, in which most may be regulated by these seven TFs (Figure 2E).

### Phloem cells are involved in flowering, nutrient transport, and stress responses

In this study, three phloem cell types (PP, CCs, and SEs) formed separate cell clusters (Figure 1A), suggesting differing transcriptome profiles. PP was specifically enriched for amino acid transmembrane transporter *BrLHT7.1*, sucrose efflux transporters *BrSWEET11.3* and *BrSWEET12.1*, phloem membrane protein *BrNHL26.3*, and nodulin MtN21-like transporter family protein *BrUMAMIT31.1* (Figure 2F and Table S4). Among them, *SWEET11*, *SWEET12*, and *NDR1/HIN1-LIKE 26* (*NHL26*) were involved in sugar export [29] [53]. To confirm the tissue localization of these genes, two genes ((*BrSWEET12* (*Bra009700*) and *CALLOSE SYNTHASE 8* (*CASL8*, *Bra027328*)) were selected for RNA in situ hybridization. *BrSWEET12* and *BrCASL8* were mainly expressed in PP (Figure S4A and B). These results suggest PP plays an important role in sugar transport and partitioning. In addition, several stress-related genes and the flowering gene *BrFBH4*.*2* were preferentially expressed in PP (Figure 2F and Table S4).

CCs provide SEs with proteins and transcripts via plasmodesmata to maintain their function. In addition to photosynthesis-related genes (Table S4), many transporter-encoding genes, including sucrose transporter *BrSUC2.1*; myo-inositol transporter *BrINT4.2*; ABC transporter *BrABCG25.1*, H^+^-ATPase *BrAHA3.1* and *BrAHA3.2*; amino acid transmembrane transporters *BrAAP1.3*, *BrUMAMIT10.2*, and *BrPROT2.2*; and metal ion transporters *BrHIPP21.1* and *BrATHMP49*; were highly expressed in CCs (Figure 2G and Table S5). Moreover, three flowering-related genes, namely *BrTPS1.1*, *BrNAKR1.2*, and *BrMYR1*, as well as several stress-related genes (*BrPP2-A1.2*, *BrCYSTM12.1*, *BrCYSTM12.2*, and *BrCEPD1.2*) were specifically expressed in CCs (Figure 2G and Table S4). We performed RNA in situ hybridization to confirm the tissue localization of two genes (*BrNAKR1* (*Bra028868*) and *BrAHA3* (*Bra002733*)). *BrNAKR1* and *BrAHA3* were mainly expressed in CCs (Figure S4C and D). GO enrichment analysis showed that CCs were enriched in amine biosynthetic process and S-adenosylmethioninamine metabolic process (Table S5). KEGG enrichment analysis also showed that CCs were enriched in genes involved in arginine and proline metabolism, cysteine and methionine metabolism, and glutathione metabolism processes, such as *BrSAMDC.1*, *BrASP2.2*, and *BrSPDS3.1* (Figure 2G and Table S6). These results suggest active amino acid metabolic processes in CCs.

SEs are highly specialized cells that are joined together by perforated end walls to form a symplasmic continuum for long-distance allocation of photosynthates and signaling molecules [54]. Copper ion transmembrane transporter *BrCOPT4*, nucleobase-ascorbate transporter *BrNAT7*, zinc ion transporter *BrMTPB1.2*, sphingosine transmembrane transporter *BrACD11*, and cation exchanger *BrCCX2.1* were specifically expressed in SEs (Figure 2H and Table S4). Many cell wall metabolism genes were also specifically expressed in SEs, including callose synthases *BrGSL07.1*, *BrGSL07.2*, and *BrGSL10.2*; 1,3-beta-glucan synthase *BrGSL03*; pectin methylesterase inhibitors *BrPMEI-PME22.1*, *BrTBL22.1*, and *BrGH9B1.1*; and sucrose synthase *BrSUS5*. In addition, SEs were also specifically enriched in flowering (*BrMYR2*, *BrEFM.2*, *BrGATA18.1*, and *BrGATA20.1*) and stress-related genes (*BrPP2-A9*, *BrMLO13.1*, *BrMLO13.2*, *BrLOX5.1*, *BrLOX5.2*, *BrIAN13*, *BrRTM1*, *BrRTM2.1*, and *BrRTM2.2*) (Figure 2H and Table S4). RTM1 and RTM2 have been shown to limit the ability of the tobacco etch potyvirus to travel long distances in *Arabidopsis* [55]. Several early nodulin-like protein-encoding genes, including *BrENODL9.1*, *BrENODL9.2*, *BrENODL9.3*, *BrENODL18.3*, and *BrENODL20*, were specifically expressed in SEs (Figure 2H). These findings imply that SEs are essential for transport, flowering, and biotic stress responses.

These results suggest that all three types of phloem cells are involved in nutrient transport, flowering, and stress responses, but they have different functional focuses. PP and CC are mainly involved in the transmembrane transport of sugars. CC is an important site for amino acid synthesis, while cell wall thickening is present in SE cells. In addition, PP, CC, and SE cells may be synergistically involved in flowering and stress response processes.

### Auxin and ethylene are involved in the early development of adaxial vascular cells

**Figure 3:**
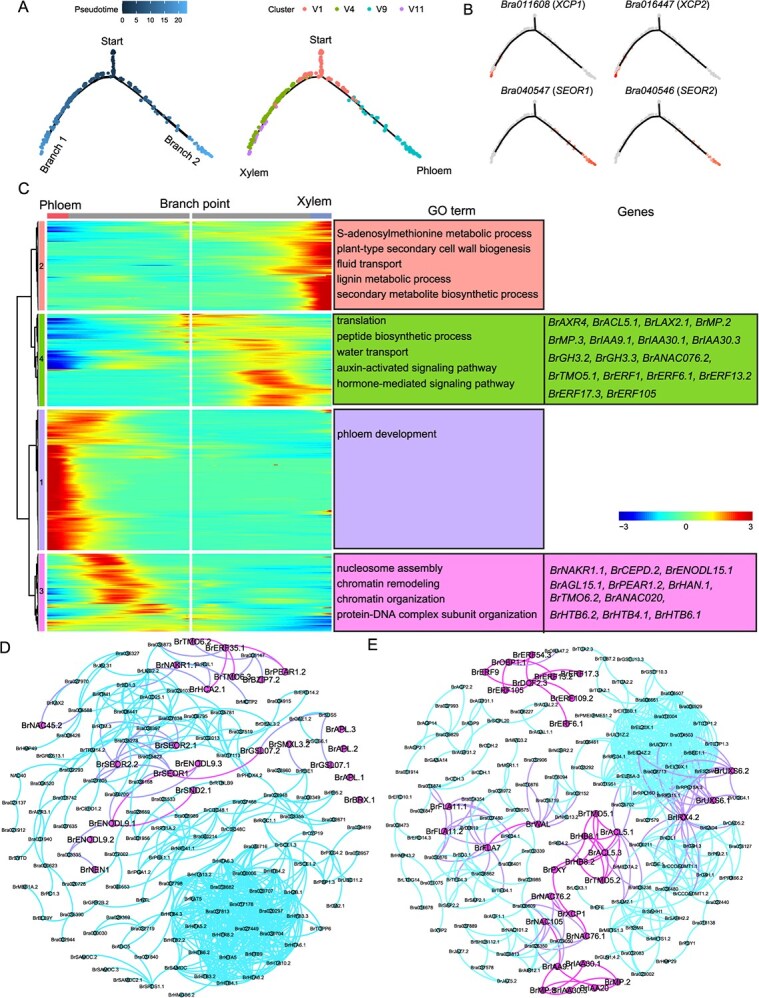
Pseudo-time analysis of adaxial and abaxial vascular cells. **(A)** Monocle 2 analysis showing the differentiation trajectory of xylem and phloem. ‘Start’ indicates the beginning of pseudo-time. Dots denote single cells. **(B)** Expression patterns of TE marker genes (*XCP1* and *XCP2*) and SE marker genes (*SEOR1* and *SEOR2*) along pseudo-time. **(C)** Heat map showing the expression characteristics of dynamically expressed genes along pseudo time. The branch point (shown in the middle of the heat map) is the beginning of pseudo-time. Both sides of the heat map represent the endpoints of pseudo-time. **(D)** Putative protein–protein interaction network of these genes from groups 1 and 3 using STRING. The red dots represent genes whose orthologs in *Arabidopsis thaliana* are involved in phloem development. **(E)** Putative protein–protein interaction network of genes from groups 2 and 4 using STRING. The red dots represent genes whose orthologs in *A. thaliana* are involved in xylem development.

The adaxial xylem and abaxial phloem were generated by procambium through cell proliferation and differentiation [56]. To infer the differentiation process from the procambium to the xylem and phloem, clusters V1, V4, V9, and V11 were selected to perform pseudo-time analysis. The cells in cluster V1 were distributed at the beginning of the trajectory and then differentiated into two distinct cell types along two branches (Figure 3A). Clusters V4 and V11 were distributed along branch 1, and cluster V9 was distributed along branch 2. Branches 1 and 2 represented the differentiation trajectories of the xylem and phloem, respectively (Figure 3A). In addition, TE (*XCP1* and *XCP2*) and SE marker genes (*SEOR1* and *SEOR2*) were specifically expressed at the ends of branches 1 and 2, respectively, supporting the above results (Figure 3B).

We next identified differentially expressed genes (DEGs) along the trajectory using the ‘BEAM’ function in Monocle 2. A total of 762 genes were identified (*q*-value < 1e-4) and divided into four groups using hierarchical clustering analysis (Table S7 and Figure 3C). Most of the genes in group 3 were first upregulated and then downregulated along the phloem trajectory, but remained lowly expressed in the pre-branch and xylem trajectories (Figure 3C). The genes in group 1 maintained low expression levels in the pre-branch and xylem differentiation trajectory; however, their expression was increased with phloem differentiation and reached a maximum at the end of the trajectory (Figure 3C). The dynamic expression of the two groups of genes along the phloem differentiation trajectory implies their role in phloem differentiation. Similarly, these genes from groups 4 and 2 were sequentially highly expressed along the xylem differentiation trajectory, indicating their roles at the early and late stages of xylem differentiation, respectively (Figure 3C).

To elucidate differentiation regulatory processes, we focused on gene sets involved in the early stages of differentiation, namely groups 3 and 4. Group 3 contained 148 genes, such as *BrNAKR1.1*, *BrCEPD.2*, and *BrENODL15.1* (Table S7). Of these 148 genes, 17 encoding TFs, including *BrAGL15.1*, *BrPEAR1.2*, *BrTMO6.2*, *BrHAN.1*, and *BrANAC020*, were identified (Figure 3C). Many genes encoding histones, such as *BrHTB6.2*, *BrHTB4.1*, and *BrHTA6.1*, were also enriched in group 3 (Table S7). GO enrichment analysis showed that group 3 was mainly enriched in ‘nucleosome assembly’ and ‘chromatin remodeling’ (Figure 3C). This implies remarkable changes in chromatin opening regions at the early stage of phloem differentiation. Protein–protein interaction analysis of the genes from groups 1 and 3 revealed a protein interaction network (Figure 3D). Some key TFs and proteins involved in phloem development, including BrTMO6.2, BrTMO6.3, BrPEAR1.2, BrBZIP7.2, BrHCA2.1, BrENODL9.1, BrENODL9.2, BrENODL9.3, BrSND2.1, BrNEN1, BrSEOR1, BrSEOR2.1, BrSEOR2.2, BrGSL07.1, BrGSL07.2, BrNAC45.2, and BrSMXL3.2 formed an interaction network. However, BrAPL.1, BrAPL.2, BrAPL.3, and BrBRX.1 were not in this network.

Unlike group 3, group 4 was enriched in many genes related to the auxin pathway, including auxin influx transporters encoding genes *BrAXR4*, *BrACL5.1*, *BrACL5.2*, and *BrLAX2.1* and auxin signaling genes *BrMP.2*, *BrMP.3*, *BrIAA9.1*, *BrIAA30.1*, *BrIAA30.3*, *BrGH3.2*, and *BrGH3.3* (Table S7). Furthermore, 36 TF-encoding genes, including *BrANAC076.2*, *BrMP.2* and *BrTMO5.1*, were identified in group 4. Of these 36 genes, seven were ethylene response factors: *BrERF6.1*, *BrERF9*, *BrERF13.2*, *BrERF17.3*, *BrERF54.3*, *BrERF105*, and *BrERF109.2* (Figure 3C). Group 4 was mainly enriched in ‘peptide biosynthetic process’, ‘translation’, ‘regulation of gene expression’, ‘auxin-activated signaling pathway’, and ‘response to hormone’ (Figure 3C). Protein–protein interaction analysis of the genes from groups 2 and 4 revealed two major protein interaction networks (Figure 3E). Some key TFs and proteins were involved in xylem development, including BrTMO5.1, BrTMO5.2, BrACL5.1, BrACL5.3, BrHB8.1, BrHB8.2, BrPXY, BrNAC105 (BrVND3), BrMP.2, BrMP.3, BrNAC76 (VND2), BrXCP1, BrIAA30, BrIAA20, and BrIAA9.1. Additionally, lignin synthesis-related proteins (BrIRX4.2, BrUXS6, BrWAL, and BrFLA7) were also part of this network. In addition, ethylene response factors (BrERF6.1, BrERF9, BrERF13.2, BrERF17.3, BrERF54.3, BrERF105 and BrERF109.2) and two DOF TFs (BrDOF2.3 and BrOBP1.1) formed an interaction network. These results show the important role of auxin and ethylene in xylem differentiation. Collectively, our results reveal insights into the early differentiation of xylem and phloem and provide data resources and candidate gene sets for future research.

### Reconstruction of CCs and SEs differentiation trajectory

**Figure 4:**
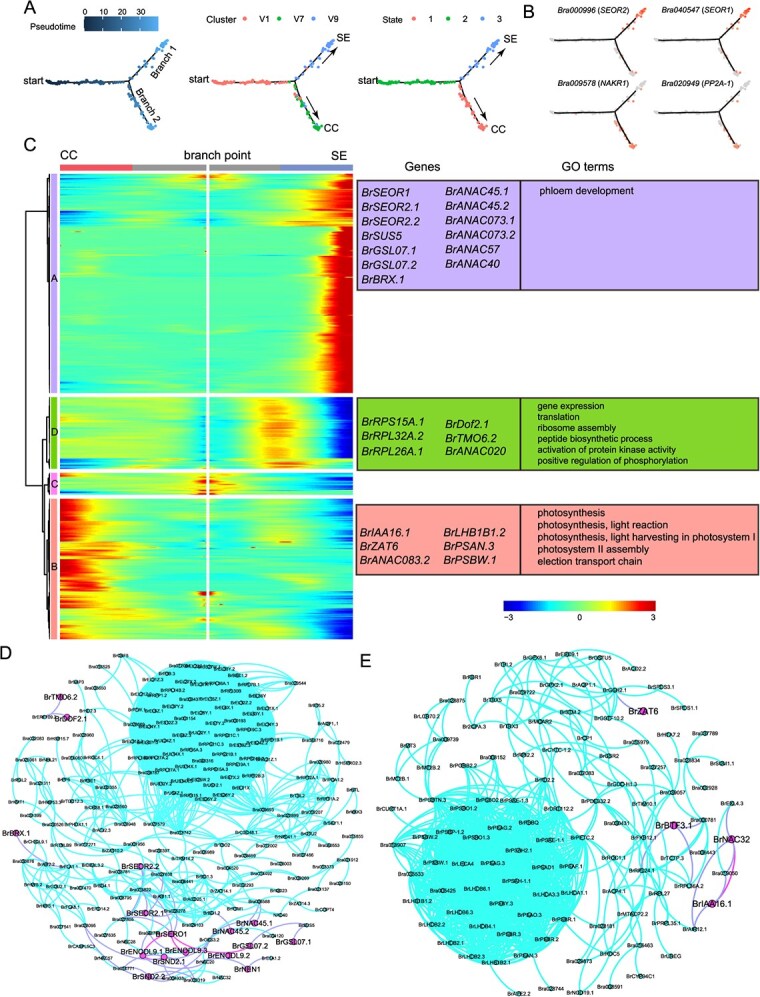
Reconstruction of the CC and SE differentiation trajectories. **(A)** Monocle 2 analysis showing the SE and CC differentiation trajectories, colored by pseudo-time, cell state, and cell cluster. ‘Start’ represents the beginning of the cell differentiation trajectory. Dots denote single cells. SE, sieve element; CC, companion cell. **(B)** Expression characteristics of important regulatory genes involved in SE and CC differentiation over pseudo-time. **(C)** Expression characteristics of dynamically expressed genes along pseudo time. The branch point is the beginning of pseudo-time. Both sides of the heat map are at the end of pseudo-time. **(D)** Putative protein–protein interaction network of genes from groups A, C, and D using STRING. The red dots represent genes whose orthologs in *A. thaliana* are involved in SE development. **(E)** Putative protein–protein interaction network of genes from groups B and C using STRING. The red dots represent the TFs.

To infer the differentiation of CC and SE, clusters V1, V7, and V9 were extracted for pseudo-time analysis. The cells in cluster V1 were distributed along the ‘start’ of the trajectory and then differentiated to produce two branches. Clusters V9 and V7 were distributed along branches 1 and 2, respectively, suggesting that branches 1 and 2 represent the differentiation trajectories of SE and CC, respectively (Figure 4A). In addition, SEs (*SEOR1* and *SEOR2*) and CC marker genes (*PP2A-1* and *NAKR1*) were specifically expressed in cells of branches 1 and 2, respectively (Figure 4B).

We identified 645 DEGs across pseudo-time (*q*-value < 1e−4, Table S8) and divided them into four groups. The genes in group A were lowly expressed in the pre-branch and CC differentiation trajectory; however, they increased with SE differentiation and reached their maximum at the end (Figure 4C). However, the majority of genes in group B were gradually upregulated along the CC differentiation trajectory but maintained a low expression level in the SE trajectory (Figure 4C). The genes in group C were only highly expressed at the beginning of the trajectory and were then sharply downregulated as the cells differentiated into CC or SE (Figure 4C). The genes in group D showed low expression in the pre-branch and CC trajectories but a sharp increase near the branchpoint, followed by a decrease in the SE trajectory. These results implied that groups A and D played a role in SE differentiation, while group B was involved in CC differentiation.

Group D was enriched with many genes related to ‘gene expression', ‘ribosome assembly', ‘peptide biosynthetic process’, ‘activation of protein kinase activity’, and ‘positive regulation of phosphorylation’, such as *BrRPS15A.1*, *BrRPL32A.2*, and *BrRPL26A.1* (Table S8 and Figure 4C). Three genes encoding TFs (*BrDof2.1*, *BrTMO6.2*, and *BrANAC020*) were identified in group D. TMO6, which encodes a PEAR protein, promotes phloem differentiation by transcriptional activation of APL [57]. *Dof2.1* is involved in early leaf vein development [58]. In addition, group A contained many genes that may be involved in SE development, such as *BrSEOR1*, *BrSEOR2.1*, *BrSEOR2.2*, *BrSUS5*, *BrGSL07.1*, *BrGSL07.2*, and *BrBRX.1*. In *Arabidopsis*, *AtSEOR1* and *AtSEOR2* are necessary for SE protein filament formation [38]. Marhava et al. (2018) found that *BRX* is involved in SE differentiation by modulating the auxin flux [59]. In addition, 21 genes encoding TFs, including *BrANAC045.1*, *BrANAC045.2*, *BrANAC073.1*, *BrANAC073.2*, *BrANAC057*, and *BrANAC040*, were identified in group A. GO enrichment analysis revealed that group A was enriched in ‘phloem development’ processes (Figure 4C). Protein–protein interaction analysis of the genes from groups A, C, and D revealed a protein interaction network (Figure 4D). Some key TFs and proteins involved in SE development, including BrENODL9.1, BrENODL9.2, BrENODL9.3, BrSND2.1, BrSND2.2, BrNEN1, BrSEOR1, BrSEOR2.1, BrSEOR2.2, BrGSL07.1, BrGSL07.2, BrNAC45.1, BrNAC45.2, and BrBRX.1 formed an interaction network.

Group B included many genes involved in ‘photosynthesis’ and ‘light reaction’ (Figure 4C). In addition, group B also contained eight TFs, such as *BrIAA16.1*, *BrZAT6*, and *BrANAC083.2* (Figure 4C). Protein–protein interaction analysis of the genes from groups B and C revealed a protein interaction network (Figure 4E). This network contained many proteins involved in light reactions, such as BrPSBW.1, BrPSBO1.2, and BrPSBQ, which exhibited close protein interactions. Furthermore, this network contained four TFs: BrIAA6.1, BrNAC32, BrBTF3.1, and BrZAT6. Overall, we identified many candidate genes involved in or regulating SE and CC differentiation.

### Subgenome asymmetric gene expression in the leaf vasculature at single-cell resolution

**Figure 5:**
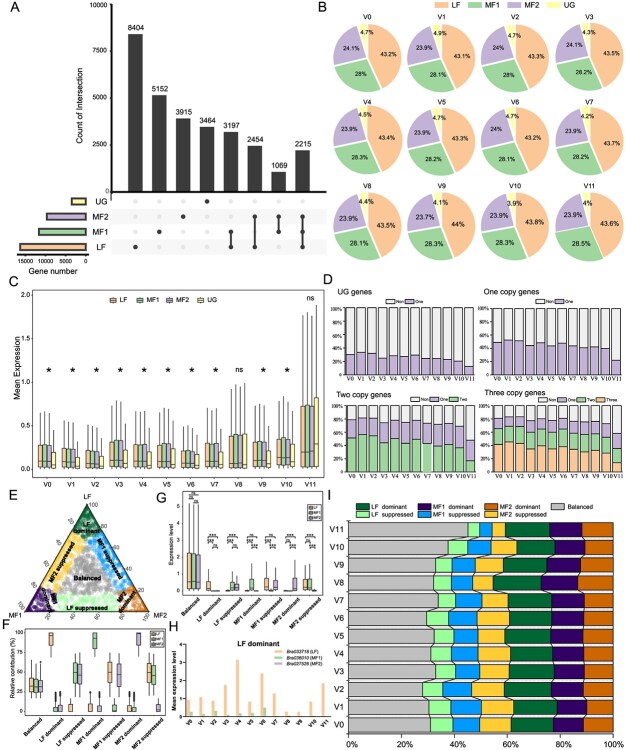
Subgenome asymmetric gene expression in leaf vasculature cells. **(A)** Gene number in each subgenome. The lines between the dots indicate the genes that are orthologous between the subgenomes. **(B)** Percentage of expressed genes from every subgenome in each cluster. **(C)** Average expression level of the genes from different subgenomes in each cell cluster. * represents *P* < 0.05 (one-way ANOVA, α = 0.05); ns represents *P* > 0.05. **(D)** Percentage of expressed genes in different cell clusters for UG genes, monads, homoeologous duplets, and homoeologous triad genes. **(E)** Plot showing the relative expression characterization of 2215 group triads in cluster V0. Each circle represents a gene triad with an LF, MF1, and MF2 coordinate composed of the relative contribution of each homeolog to the overall triad expression. Triads in vertices correspond to categories where a single subgenome is dominant, whereas triads near the edges and between vertices correspond to suppressed categories. Balanced triads are displayed in gray. **(F)** Box plots represent the relative contribution of each subgenome based on a triad assigned to the seven categories. **(G)** Box plots representing the gene expression level of each subgenome based on a triad assigned to the seven categories. ^***^ represents *P* < 0.001 (one-way ANOVA, α = 0.05); ns represents *P* > 0.05. **(H)** Gene expression level of a group triad belonging to the LF dominant category. (I) Percentage of triads in each category with homeolog expression bias across each cluster.

*Brassica rapa* includes three subgenomes (LF, MF1, and MF2) that differ in gene density and expression level [60]. LF subgenome contains 16270 genes (39.7% of the whole genome); MF1 contains 11633 genes (29.4%); MF2 contains 9653 genes (23.5%); and the other genes (3464 genes) were ungrouped genes (UG) (8.4%) [7] (Figure 5A). There were 22495, 23980, 23515, 20339, 22141, 20325, 22018, 20052, 19038, 19546, 18171, and 11047 genes expressed in clusters V0–V12, respectively. The expression ratio/density of the subgenomes was in the following order: LF > MF1 > MF2 > UG. In each cell population, 43% of the expressed genes were from the LF subgenome, 28% from MF1, 24% from MF2, and 4% from UG (Figure 5B). The average expression levels of genes were similar among LF, MF1, and MF2 within each cluster (Figure 5C). Except for clusters V8 and V11, the average expression levels of genes from LF, MF1, and MF2 were significantly higher than those from UG, respectively (Figure 5C).

Compared to *Arabidopsis*, a large number of genes in *B. rapa* has homoeologous genes, namely 3464 UG, 17471 monads, 6720 homoeologous duplets, and 2215 homoeologous triads, due to the additional genome triplication (Figure 5A). We compared the number of expressed genes of the four gene types in each cell cluster and found that the expressed proportion of UG genes was the lowest (20%–30%) (Figure 5D). In terms of homoeologous genes, the highest proportion of genes expressed was homoeologous triads, followed by duplet genes and monad genes (Figure 5D). To explore gene differentiation, we focused on homoeologous triads and divided them into seven categories: balanced, LF dominant, MF1 dominant, MF2 dominant, LF suppressed, MF1 suppressed, and MF2 suppressed, based on the expression patterns described by Ramirez-Gonzalez et al. [61](Figure 5E, S3A and table S9). The relative contribution and gene expression levels of each homoeologous triad from the seven categories are shown in Figure 5F and 5G, respectively. For example, *Bra033718*, *Bra036010*, and *Bra027526* were located in LF, MF1, and MF2 subgenome, respectively, and they were orthologs to *AT5G44070*, which encodes phytochelatin synthase. Compared to *Bra036010* and *Bra027526*, *Bra033718* had a much higher expression level; thus, this triad was classified as LF dominant (Figure 5H). The examples of the other five categories (MF1 dominant, MF2 dominant, LF suppressed, MF1 suppressed, and MF2 suppressed) are also shown in Figure S5B. Most homoeologous triads in each cell cluster showed asymmetric gene expression. Among the cell types, the mean proportion of LF dominant was the highest (16.46%), followed by MF1 dominant (12.06%), MF2 dominant (11.31%), LF suppressed (7.24%), MF1 suppressed (9.33%), and MF2 suppressed (10.41%) (Figure 5I and Figure S5). These results show that there is also subgenome asymmetric gene expression in different leaf vascular cells at the single-cell level, suggesting that homoeologous genes undergo differentiation after genome triplication.

## Discussion

*Brassica rapa* is a species that encompasses many leafy vegetables that exhibit extreme leaf morphological variations, e.g. heading Chinese cabbages and non-heading leafy vegetables, including pak choi, Wutacai, and Mizuna. Current methods of morphological observation and genetic and domestication selection research have shown that leaf curvature or heading in *Brassica* crops is associated with changes in leaf adaxial-abaxial patterning [7,11,62–67]. The adaxial–abaxial nature of the leaf is reflected in the adaxial–abaxial patterning of vascular tissue, mesophyll cells, and the epidermis. Guo et al. described the differentiation between palisade mesophyll and spongy mesophyll cells in *B. rapa* using scRNA-seq [29]. However, little is known about the differentiation and development of vascular tissue in *B. rapa* leaves. The heading-related gene *BrOPS* and two candidate genes (*BrBRX.1* and *BrBRX.2*) are preferentially expressed in leaf vasculature [7,11,68]. Therefore, distinguishing between adaxial and abaxial cell types in leaf vasculature and describing their differential gene expression are necessary for exploring the leaf development and morphogenesis mechanisms of *Brassica* vegetables. Here, we present a single-cell expression atlas of *B. rapa* leaf vasculature. The leaf vasculature was composed of highly heterogeneous cells with 12 transcriptionally distinct cell clusters. Seven cell types were successfully identified, namely XP, TE, PP, SE, CC, PC^XP^, and PCs, that covered the known main cell types of the leaf vasculature. However, several cell clusters remained undefined in our data, indicating that the current knowledge of leaf vein cell types is inadequate. Spatial transcriptome may will help to assign undefined clusters in the future.

Cell type identification in the leaf vasculature allowed us to explore their transcriptome characteristics. The xylem included XPs and TEs, which exhibited distinct transcriptome profiles. XP was not only enriched with many transporter-encoding genes but also exhibited an active expression pattern of amino acid metabolism genes. Unlike XP, TEs were mainly enriched for genes related to cell wall biosynthesis. There were different transcriptome characteristics among the different cell types in the phloem population. PP was specifically enriched in the sucrose efflux transporter-encoding genes *BrSWEET11.3* and *BrSWEET12.1*, similar to *Arabidopsis* [3]. CC was enriched in a large number of amino acid metabolism genes, indicating its active amino acid metabolism profile. Similar to TE, SE was enriched in many cell wall biogenesis genes. In addition, PP, CC, and SE may be synergistically involved in flowering and stress response processes.

**Figure 6:**
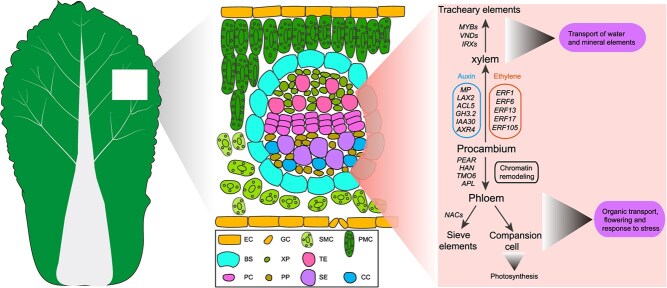
Hypothetical regulatory model for vascular tissue differentiation in Chinese cabbage leaves. Auxin and ethylene were involved in the differentiation process from procambium to xylem. Xylem is involved in water and mineral element transport. The TFs MYBs and VNDs, as well as *IRXs* genes regulate and participate in TE formation. Phloem (including SE and CC) is mainly involved in flowering, organic transport, and stress response. TF encoding genes *PEAR*, *HAN*, *TMO6*, and *APL* are involved in the differentiation process from the procambium to the phloem, whereas many NAC TFs regulate SE formation. EC, epidermis; GC, guard cell; PMC, palisade mesophyll cell; SMC, spongy mesophyll cell; BS, bundle sheath; XP, xylem parenchyma; TE, tracheary elements; PC, procambium cell; PP, phloem parenchyma; SE, sieve elements; CC, companion cells.

We explored the differentiation trajectories of leaf vascular cells by pseudo-time analysis. A model was used to describe the potential roles and functions of the genes during vascular tissue differentiation in Chinese cabbage (Figure 6). In addition to many genes involved in auxin transport and signaling, we identified several ethylene response factors (*BrERF1*, *BrERF6.1*, *BrERF13.2*, *BrERF17.3*, and *BrERF105*) in the early stage of xylem differentiation (Figure 6). In *Arabidopsis*, ethylene treatment causes a decrease in secondary xylem [69]. Etchell et al. found that *ERF1*, *ERF018*, and *ERF109* were involved in cambial cell division in *Arabidopsis* [70]. Miller et al. found that ethylene induces xylem differentiation in *Lactuca* [71]. In hybrid aspen, ethylene-induced PtERF85 coordinates the xylem cell expansion process as well as secondary cell wall formation [72]. This implies that ethylene and auxin play a role in xylem differentiation. The TFs MYBs and VNDs as well as *IRXs* genes may regulate and participate in TE formation in *B. rapa*.

Many genes related to ‘nucleosome assembly’ and ‘chromatin organization’ were highly expressed in the early stages of phloem differentiation (Figure 6). Furthermore, we inferred the differentiation trajectories of CC and SE. We found that eight NAC TF-encoding genes, namely *BrNAC45.1*, *BrNAC45.2*, *BrNAC073.1*, *BrNAC073.2*, *BrNAC40*, *BrNAC57*, *BrNAC28*, and *BrNAC53.2*, were enriched in the SE differentiation process, suggesting the important role of NAC transcription factors in SE differentiation (Figure 6). In *Arabidopsis*, NAC45 regulates the SE enucleation process [73]. Kim found that *NAC73* was involved in SE development in *Arabidopsis* roots [74]. Many genes involved in ‘photosynthesis’ and ‘light reaction’ were enriched in the CC differentiation trajectory, which was consistent with CC being capable of photosynthesis (Figure 6).

Polyploidization is widespread and a key source of motivation for plant evolution. The asymmetric evolution of subgenomes is often obvious in ancient polyploidized genomes. The divergence of homoeologous genes generated by polyploidizations fueled trait innovation [75–77]. Compared to *Arabidopsis*, Chinese cabbage underwent additional triploidization, containing three subgenomes (LF, MF1, and MF2) [28], which were asymmetric in gene density and expression characterization. The LF subgenome retained most genes, followed by MF1 and then MF2. The genes in the LF subgenome were dominantly expressed over those in MF1 or MF2, while the genes in MF1 were slightly dominantly expressed over those in MF2 [78]. Sun et al. found that genes from the LF subgenome were dominantly expressed over those from the MF1 and MF2 in different leaf cell types using scRNA-seq [27]. This is consistent with previous findings at the tissue level. Here, we found that in different vascular cells types, the largest proportion expression ratio/density of subgenomes was LF, followed by MF1 and then MF2, which is consistent with the results of Sun et al. [27]. Similar to wheat, homoeologous triad genes from the three *B. rapa* subgenomes can be categorized into seven expression patterns (balanced, LF dominant, MF1 dominant, MF2 dominant, LF suppressed, MF1 suppressed, and MF2 suppressed) based on their expression levels. However, previous studies have only focused on genes that are dominantly expressed in the tissue or cell type of Chinese cabbage, which only covers three expression patterns (LF dominant, MF1 dominant, and MF2 dominant). This study focused on all seven expression patterns and found that most of the homoeologous triad genes (~ 70%) were unbalanced among vascular cells.

In conclusion, we constructed a single-cell expression atlas of Chinese cabbage leaf vascular tissue. This is the first report on the gene expression of each cell type in the leaf vasculature of Chinese cabbage. Different cell types showed distinct identities. XP exhibited an active amino acid metabolic profile, and TEs and SEs were enriched for many cell wall biosynthesis genes. PP was involved in sugar transport and partitioning, and CCs were an important site for amino acid metabolism. To infer the differentiation process, we reconstructed xylem and phloem differentiation trajectories using pseudo-time analysis and identified key candidate genes involved in the differentiation process, e.g. *BrPEAR1.2*, *BrTMO6.2*, *BrHAN.1*, *BrANAC020*, *BrANAC076.2*, *BrMP.2*, and *BrTMO5.1*. In addition, CC and SE differentiation trajectories were reconstructed, and candidate genes related to the differentiation process were identified. Therefore, elucidating the functions of cluster-enriched TFs will expand our understanding of the leaf vasculature development process. Our study not only provides many vascular-cell-specific marker genes but also deepens our understanding of leaf morphogenesis and development in *Brassica* vegetables.

## Materials and methods

### Preparation of protoplast suspensions and scRNA-seq library sequencing

*Brassica rapa*, variety Chiifu-401-42, was seeded in a greenhouse. Three-week-old seedlings were transplanted into the field in the plastic shed. The young rosette leaves were harvested from normally growing Chinese cabbages at the rosette stage. After removing the leaf ribs, these samples were sliced into strips and digested in digestion solution (1.5% cellulase R10, 0.4% macerozyme R10, 0.4 M mannitol, 0.1 M 4-morpholineethanesulfonic acid, 20 mM KCl, 10 mM CaCl_2_, and 0.1% bovine serum albumin) at 25 °C for 2.5 h in the dark with 45–55 rpm. The protoplasts were washed thrice with 8% mannitol at room temperature. Protoplast viability was estimated using trypan blue staining. For scRNA-seq, libraries were constructed following the Single Cell 3' Reagent Kit v2 protocol (10x Genomics, CA, USA), and then were sequenced by the Illumina sequencing platform (CapitalBio Technology Beijing Co., Ltd), producing 150 bp paired-end reads.

### The preparation and preprocessing of scRNA-seq data

To investigate leaf veins in *B. rapa* at single-cell resolution, scRNA-seq data [29] of Chinese cabbage rosette leaves from our previous study were reanalyzed. The raw scRNA-seq data were stored in the National Genomics Data Center (NGDC) database (Accession number PRJCA009630). All raw sequencing data were aligned to the Brapa_V1.5 reference genome to generate gene-cell expression matrices using Cell Ranger 3.1.0 with default parameters. DoubletFinder (v.2.0.3) [79] was used to detect doublets with the following three parameters: nExp, pN, and pK. Following the standard Seurat workflow up to the clustering stage (resolution = 0.5), cell clusters were generated. These cluster labels served as annotation data to estimate the proportion of homotypic doublets. The scale of the doublet was set to N*8*1e-6 (where N is cell number). The nExp parameter was determined based on the doublet proportion of the same type and the doublet ratio. The ‘summarizeSweep’ and ‘find.pK’ functions were used to determine the optimal pK parameter, and pN was set to 0.25. The ‘doubletFinder_V3’ function was then applied to the preprocessed Seurat data to predict doublets, using the previously determined values for nExp, pN, and pK. The cells marked as singlets were used for subsequent analysis, while the cells marked as doublets were removed. Finally, a total of 183 doublets were identified using DoubletFinder.

### Data integration and clustering

Seurat (v.4.0.3) [80] was used for subsequent clustering analysis. The low-quality cells and genes were excluded according to the following criteria: (1) cells with expressed genes fewer than 200 or over 11000 were filtered; (2) cells with mitochondrial gene percentages greater than 5% or chloroplast gene percentages greater than 30% were filtered; and (3) genes detected in a minimum of three cells were retained. Following data normalization, highly variable genes were detected using the ‘vst’ approach and 2500 features. The ‘ScaleData’ function was utilized for data scaling. The linear dimensionality reduction was carried out using the ‘RunPCA’ function with 100 principal components. Cell clusters were visualized using the ‘TSNE’ and ‘RunUMAP’ algorithms. Cluster-specific genes were identified with Wilcoxon rank sum test, ‘logfc.threshold = 0.58’, and ‘min.pct=0.25’. Owing to the obvious batch effects among the three samples, ‘RunHarmony’ (v0.1.0) was used to integrate the data and correct the batch effects [81].

### Clustering of leaf vascular cells

To explore the cellular heterogeneity of the leaf vasculature, vascular cell populations (clusters 5, 13, and 16 in Figure S2) were selected and reclustered. Raw gene matrix was imported into the Seurat package to create a new Seurat object, which was analyzed following the Seurat process described above. After removing the batch effect with R package ‘Harmony’ v1.0, we adjusted the number of principal components (npcs = 50), size (dims = 20), number of adjacent points (n.neighbors = 30), resolution = 0.7, and minimum distance (min.dist = 0.3) for dimensionality reduction and other parameters to determine the best clustering effect. Cluster-enriched genes were identified as described above.

### Identification of one-to-one orthologs between *B. rapa* and *Arabidopsis*

First, we downloaded the protein sequences of two species (*B. rapa* and *Arabidopsis*) from the The *Arabidopsis* Information Resource (TAIR) and *Brassicaceae* Database (BRAD) websites, respectively. The protein sequences of homologs between *B. rapa* and *Arabidopsis* were clustered using the OrthoMCL algorithm based on an all-against-all strategy with BLASTP (e-value: 1-e5). The outcomes were categorized into three types: one-to-one orthologs, one-to-many groups, and many-to-many groups. For cross-species scRNA-seq analysis, the one-to-one orthologs were selected, which were detailed in Table S3.

### Interspecies scRNA-seq data integration

We first constructed a single-cell transcriptome map of *Arabidopsis* leaves using a published leaf scRNA-seq dataset (accession number GSE161482). The Cell Ranger 3.0.1. was used to process the scRNA-seq sequencing data. After quality control, 6300 cells and 20290 genes were obtained. The Seurat workflow was executed following the previously described process with similar parameters. Fifteen cell clusters covering main *Arabidopsis* leaf cell types were revealed after clustering (Figure S3A and B). Among them, clusters 4, 8, and 12 belonged to vascular cells (1007 cells). They were extracted and further divided into six cell clusters, and covered the main vascular cell types (Figure S3C and D). These *Arabidopsis* leaf vascular cells were then used for integration with Chinese cabbage vein cells. Before integration, the genes in the scRNA-seq data of *B. rapa* were replaced with the one-to-one orthologs in *Arabidopsis* from Table S3. The scRNA-seq datasets integration was performed with Canonical Correlation Analysis (CCA) in Seurat. After integration, the batch effects across species were eliminated.

### Pseudo-time analysis

The differentiation trajectories of the vascular cells were reconstructed using Monocle (v.2.2.0) [82]. We extracted the raw data of specific cell clusters to analyze the developmental trajectories. The expression variation was calculated using the ‘dispersionTable’ function. Variable genes were selected according to their mean expression (mean_expression ≥ 0.1) and dispersion (dispersion_empirical ≥ 1 * dispersion_fit) to determine the developmental progression. Data dimensionality reduction was performed via the ‘DDRTree’ technique. The ‘orderCells’ function was used to infer the differentiation trajectory, which was plotted using the ‘plot_cell_trajectory’ function. The ‘BEAM’ function was utilized to identify branch-dependent genes. The expression heat maps of branch-dependent genes were plotted using the ‘plot_genes_branched_heatmap’ function.

### GO ontology and KEGG enrichment analysis

GO enrichment analysis was conducted utilizing GO database (http://geneontology.org/) with false discovery rate (FDR) correction (FDR < 0.05). In addition, these cluster-enriched genes were analyzed to identify significantly enriched metabolic pathways using the KEGG Orthology Based Annotation System (KOBAS) database with FDR correction (FDR < 0.05) [83].

### Subgenome asymmetric gene expression analysis

According to the BRAD database (http://brassicadb.cn/) [84], the genes from the different subgenomes (LF, MF1, MF2) were extracted and counted. According to the number of homeologs from every subgenome, the genes were categorized into four groups: triads that refer to 1:1:1 triads (with a single copy from each of the LF-, MF1-, and MF2-subgenomes); duplets referring to 1:1:0, 1:0:1, and 0:1:1 duplets; monads group containing genes with no homeologs (e.g. 0:0:1); and ‘UG’ containing genes that were not grouped into any one subgenome. Each homoeologous triad was used to analyze homoeologous expression bias. Following the method described in previous studies [61,85], we calculated the Euclidean distance of each triad, and classified the triads into seven categories, namely Balance, LF dominant, MF1 dominant, MF2 dominant, LF suppressed, MF1 suppressed, and MF2 suppressed.

### RNA *in situ* hybridization assay

Specific FAM (carboxyfluorescein)-labeled probes of four genes (*BrSWEET12* (*Bra009700*), *BrNAKR1* (*Bra028868*), *BrCASL8* (*Bra027328*), and *BrAHA3* (*Bra002733*)) were designed and synthesized to detect their cellular localization (Table S10). For in situ hybridization, *B. rapa* leaf slices (sampled at the rosette stage) were processed as described by Xu et al. [86].

### Analysis and visualization of potential protein–protein interaction network

First, the amino acid sequences of cluster-enriched genes and DEGs identified by pseudo-time were extracted. The potential interaction networks between these sequences were analyzed using STRING. The organisms was set to *B. rapa* subsp. *pekinensis*. Other parameters were set as default. Visualization of the potential protein–protein interaction network was performed using Gephi_v0.9.2.

## Supplementary Material

Web_Material_uhaf060

## Data Availability

The data used in this study are included in the article.
