# Supplementary figures and images for: Developmental landscape and asymmetric gene expression in the leaf vasculature of Brassica rapa revealed by single-cell transcriptome
Source: Hortic Res. 2025 Feb 26;12(6):uhaf060. doi: 10.1093/hr/uhaf060 (PMC12017798; doi:10.1093/hr/uhaf060)

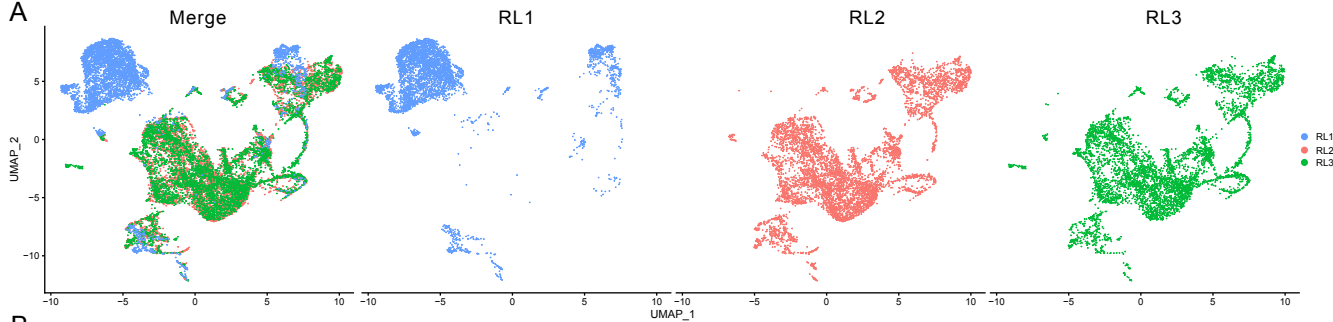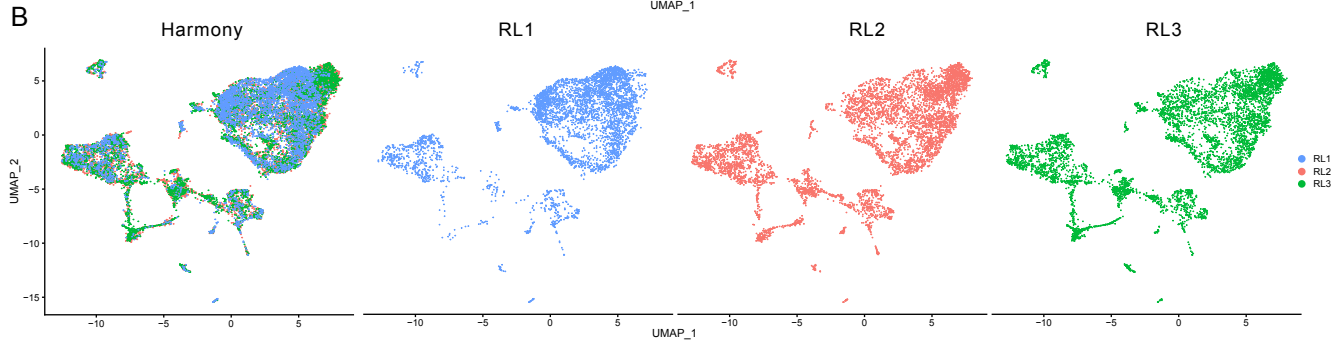

Supplement: Web_Material_uhaf060 [file web_material_uhaf060.zip › Figure S1.pdf]

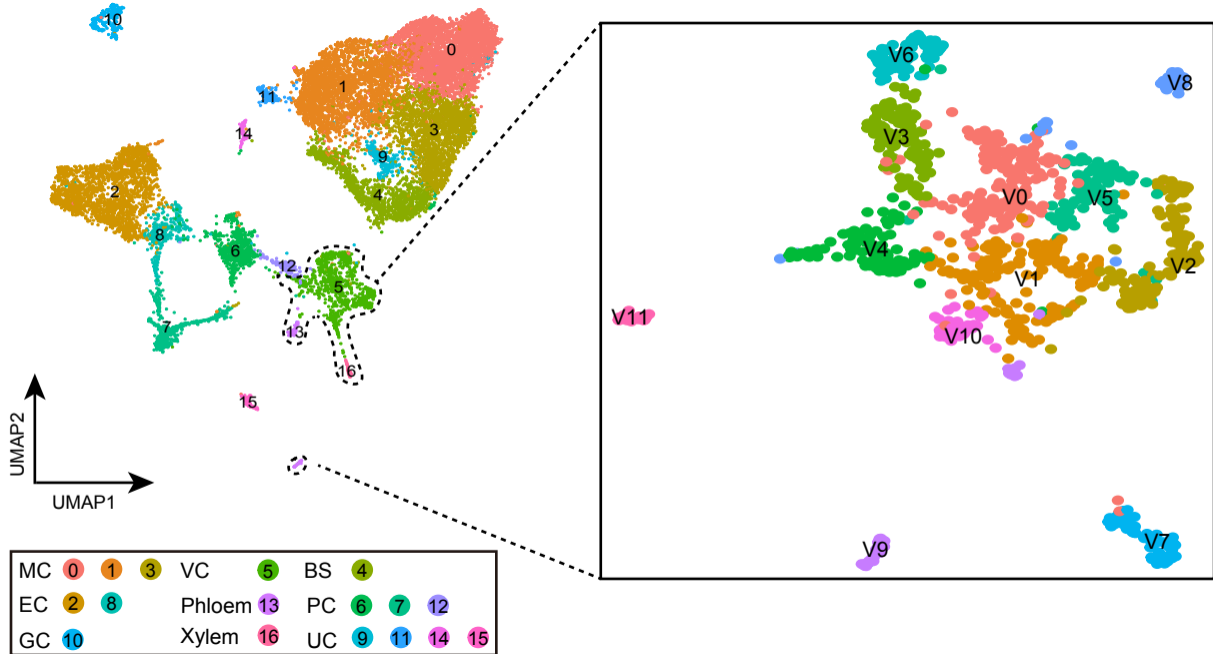

Supplement: Web_Material_uhaf060 [file web_material_uhaf060.zip › Figure S2.pdf]

A

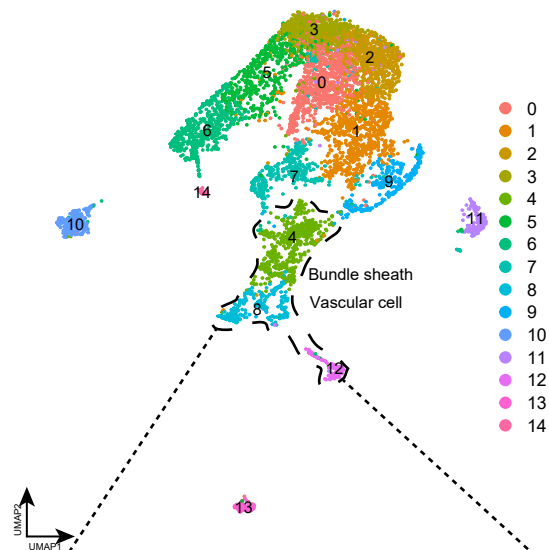

B

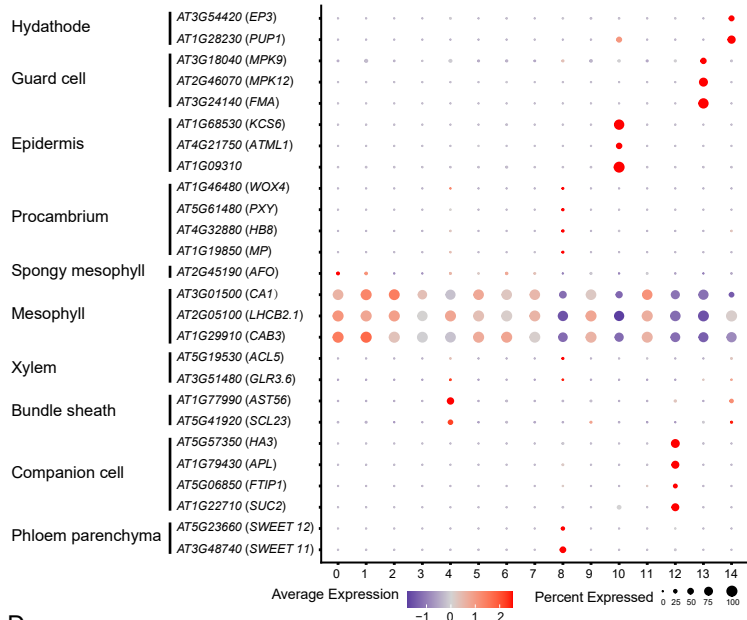

C

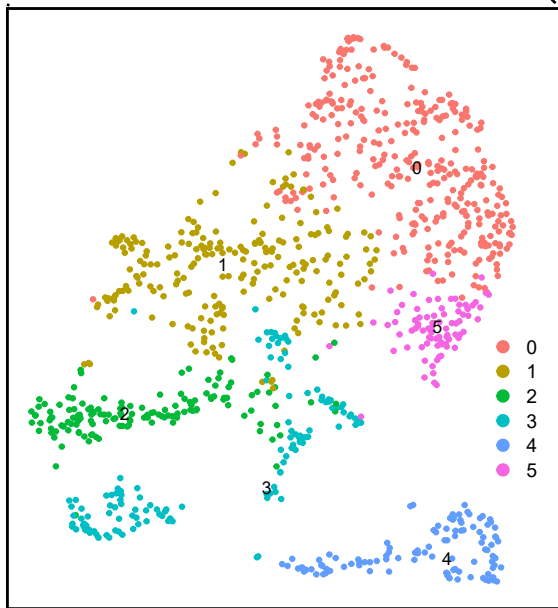

D

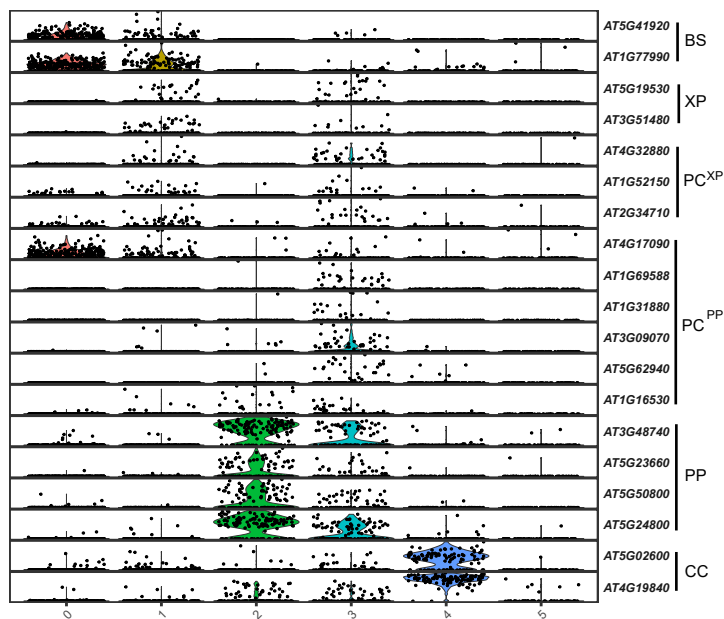

Supplement: Web_Material_uhaf060 [file web_material_uhaf060.zip › Figure S3.pdf]

A

*BrSWEET12*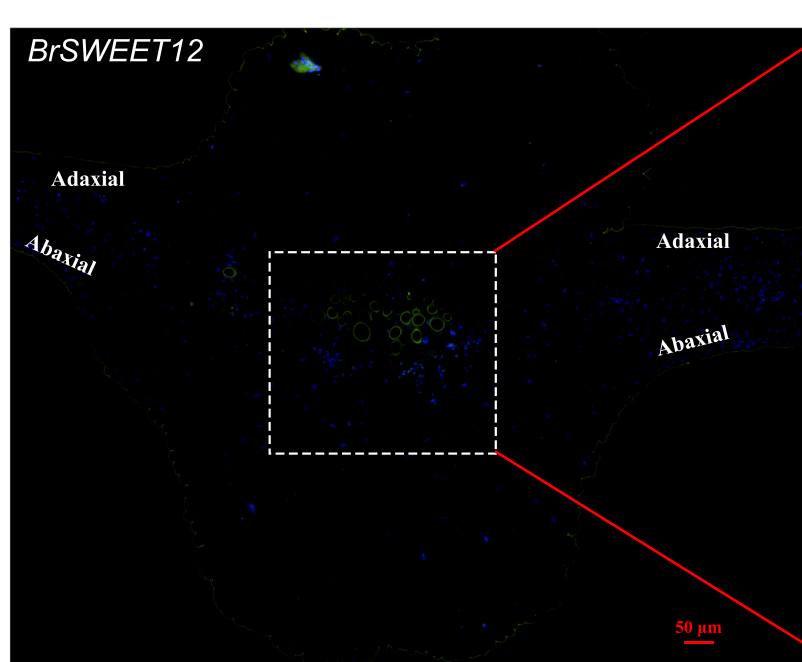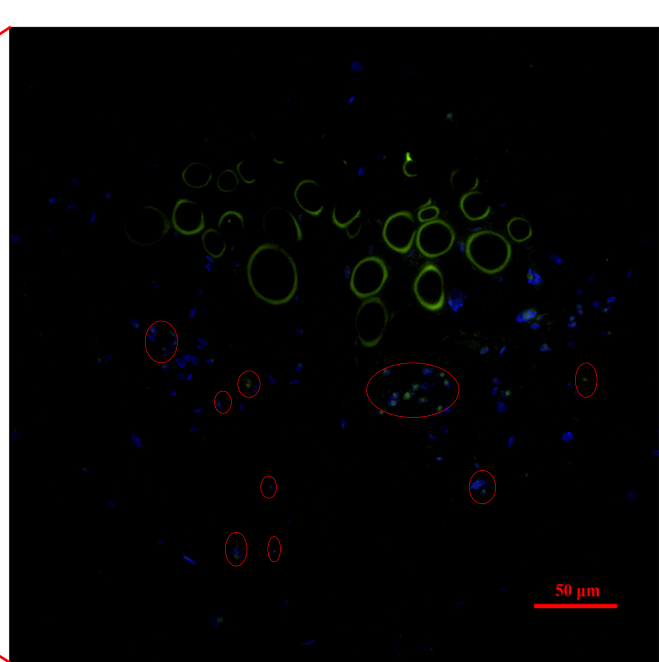

B

*BrCASL8*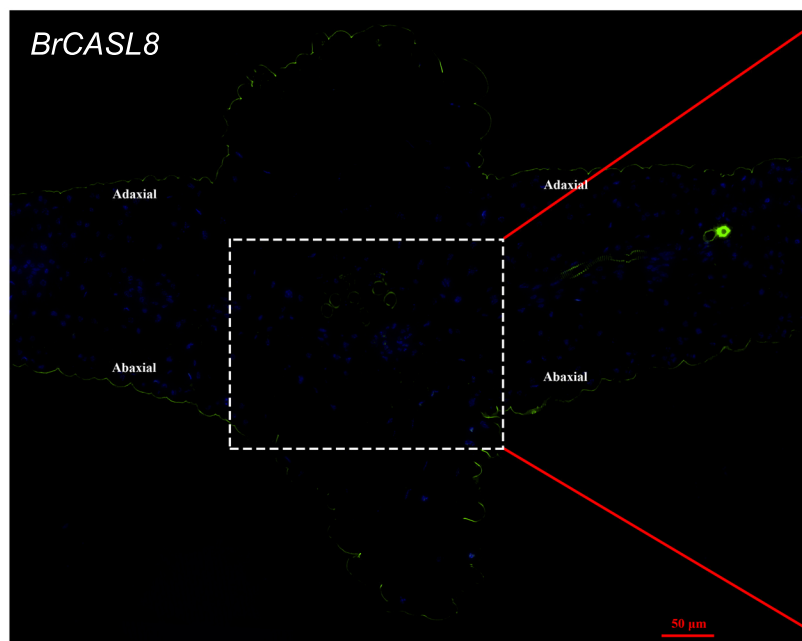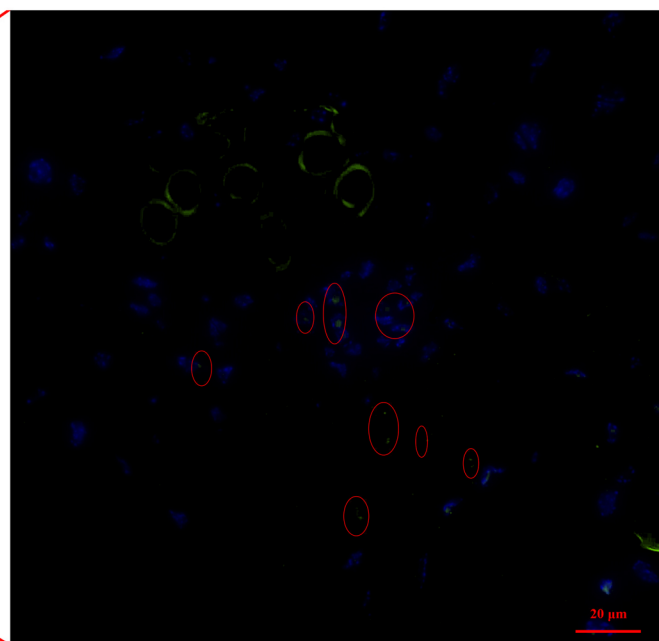

C

*BrNAKR1*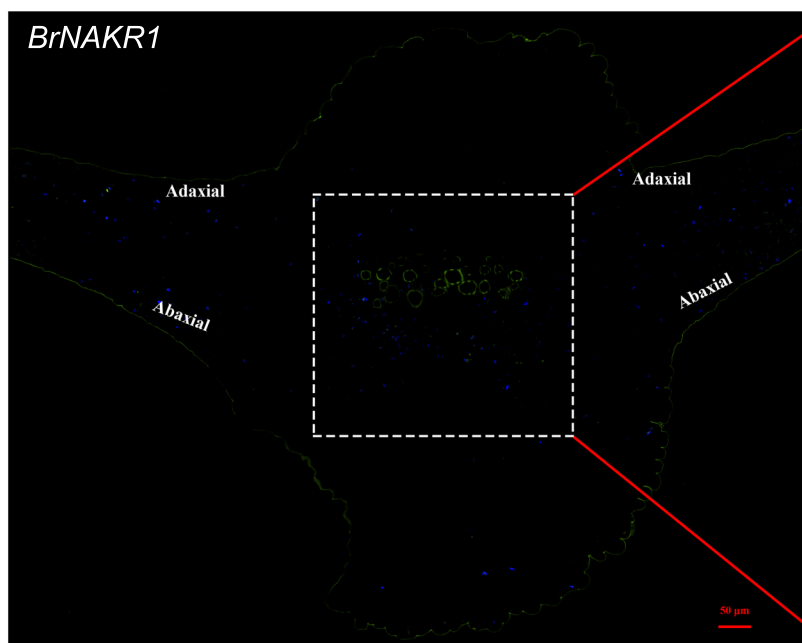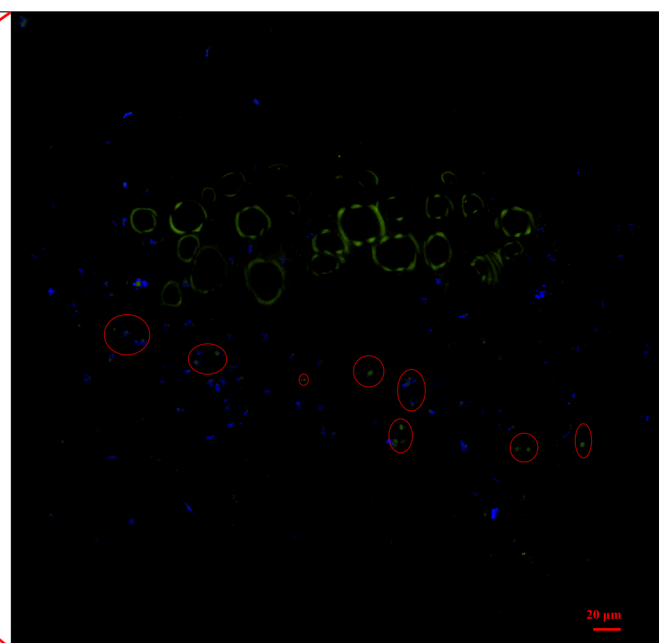

D

*BrAHA3*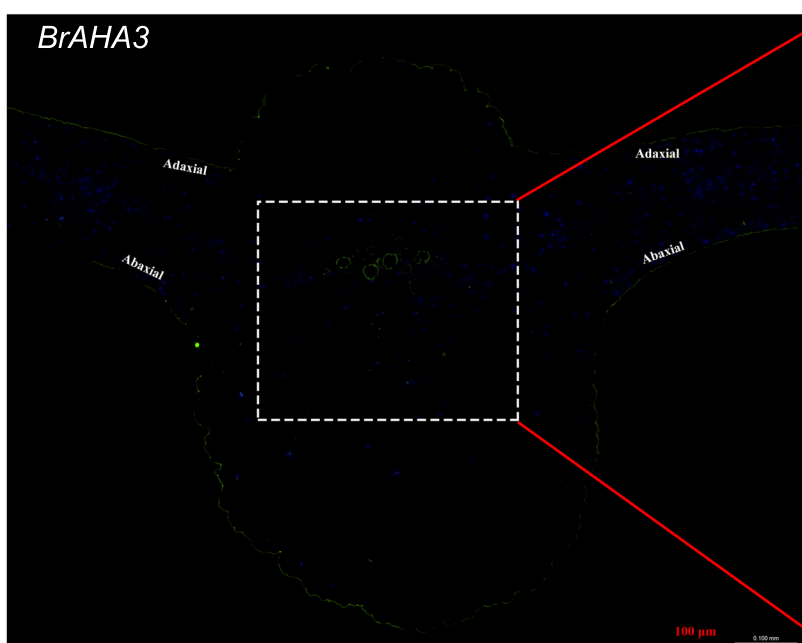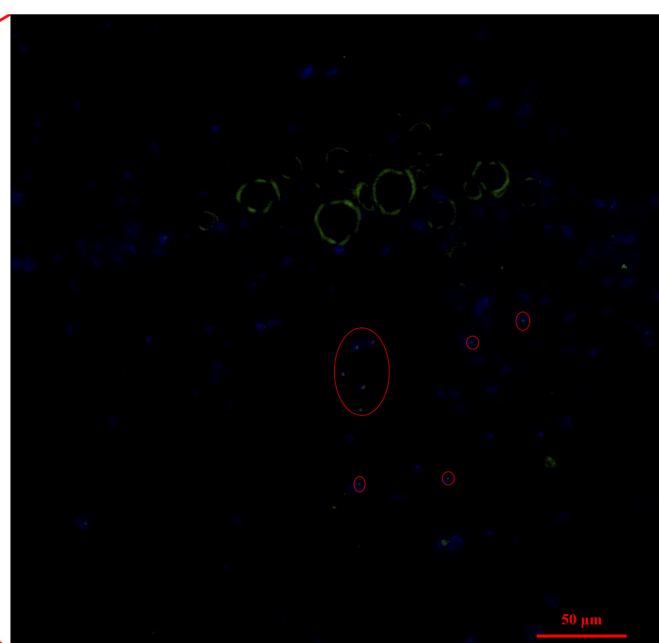

Supplement: Web_Material_uhaf060 [file web_material_uhaf060.zip › Figure S4.pdf]

**A**

● LF dominant    ● MF1 dominant    ● MF2 dominant    ● Balanced  
● LF suppressed    ● MF1 suppressed    ● MF2 suppressed

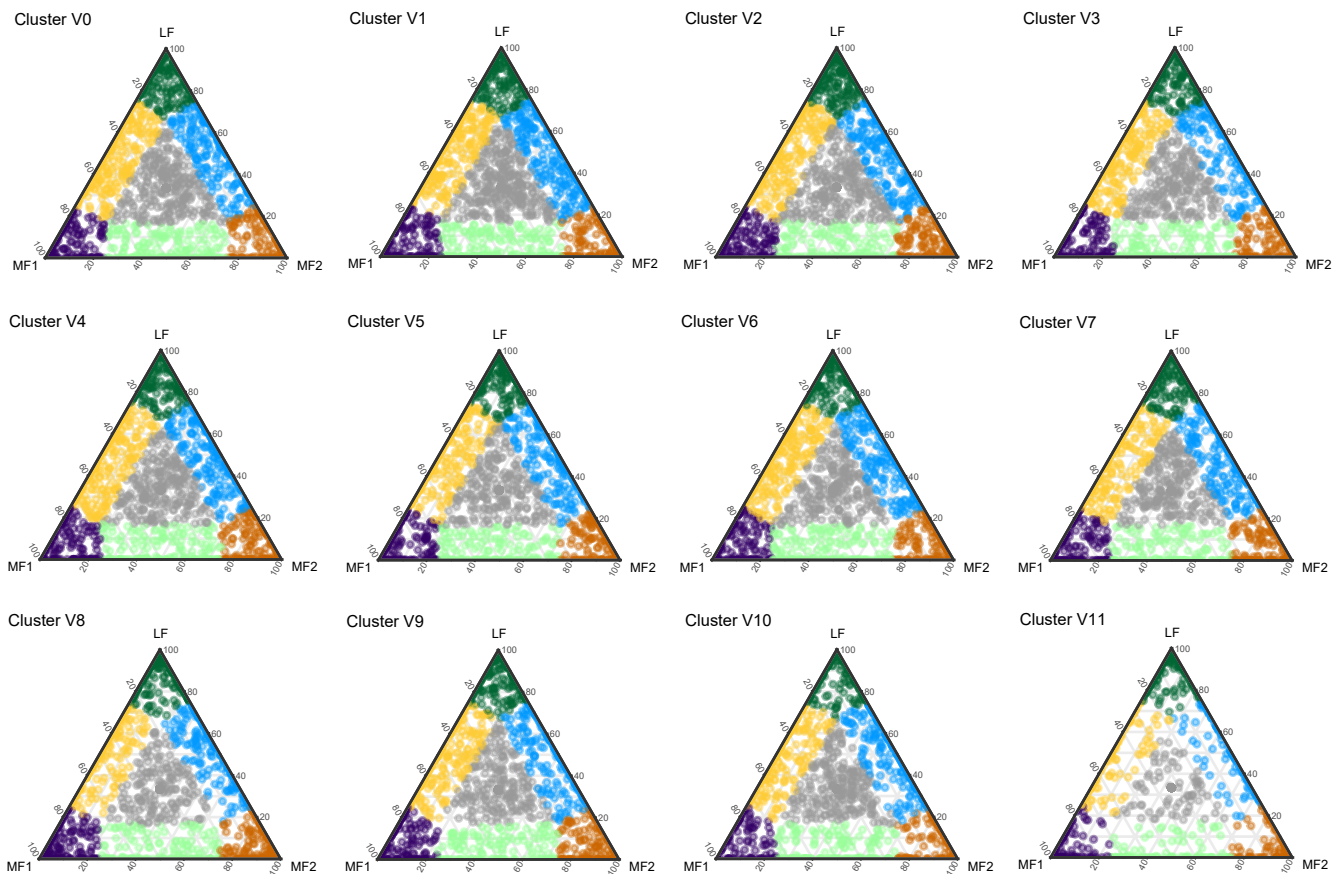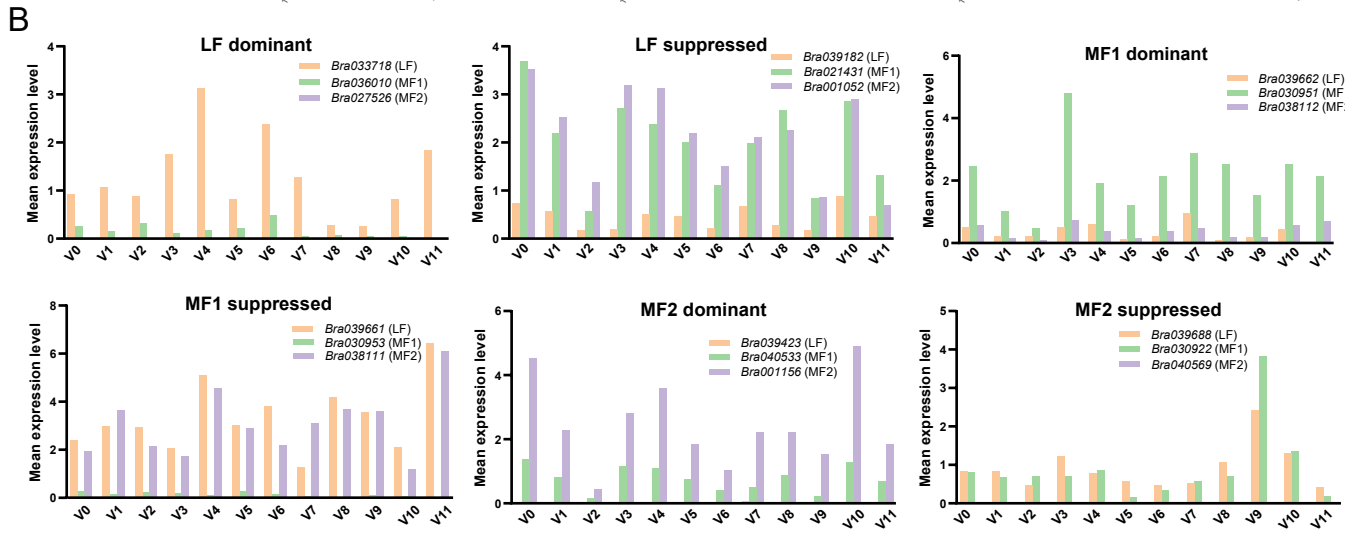

Supplement: Web_Material_uhaf060 [file web_material_uhaf060.zip › Figure S5.pdf]
